# A new patient mobilization scoring system in the ICU: what is the degree of similarity in scores between assessors in daily use?

**DOI:** 10.1186/cc11133

**Published:** 2012-03-20

**Authors:** M Vogel, CW Casteleijn, P Bruins, AJ Meinders

**Affiliations:** 1 St Antonius Ziekenhuis, Nieuwegein, the Netherlands

## Introduction

Inactivity and immobility in ICU patients have significant deleterious physiologic effects, including atelectasis, pressure ulcers, and increased susceptibility to aspiration and pneumonia. A new trend on the ICU is early mobilization of critically ill adult patients. However, evidence of when to start mobilization is missing. Casteleijn developed a new scoring system, the Patient Mobilization Frame (PMF), to improve early mobilization in the ICU. The framework is based on a multidisciplinary agreement. The aim of this study was to evaluate interobserver agreement using the PMF.

## Methods

A prospective observational study in 47 critically ill patients in the ICU was performed. The PMF categorizes patients into one of three stages of possible training using a scoring system based on 14 items. Various factors influencing individual stage are used including circulation, respiration, infection, kidney function, wounds and neurology. Stage A (critically ill) permits only passive physical examination. Whereas stage B (stable) and stage C (nearly recovered) permit (guided) active mobilization and functional training, respectively. Two staff members and one resident obtained 47 independent observation series of the PMF. All observations were at the same date and time and were compared.

## Results

Interobserver reliability of observers 1, 2 and 3 proved to be adequate. Kappa for observers 1 and 2 was 0.9. Kappa for observers 1 and 3 was 0.6. Kappa for observers 2 and 3 was 0.6. The value of kappa can range from 0 (disagreement) to 1 (perfect agreement). Kappa larger than 0.6 was regarded as substantial agreement.

## Conclusion

Casteleijn's PMF proved to be a reliable scoring system as both resident and staff members had comparable results for staging the physical abilities of the critically ill patient in the ICU.

